# GPR15 in colon cancer development and anti-tumor immune responses

**DOI:** 10.3389/fonc.2023.1254307

**Published:** 2023-11-24

**Authors:** Hong Namkoong, Bomi Lee, Gayathri Swaminathan, Seong-Joon Koh, Stephan Rogalla, Maria D. Paraskevopoulou, Jay Tang, David Mikhail, Laren S. Becker, Aida Habtezion

**Affiliations:** ^1^ Division of Gastroenterology and Hepatology, Department of Medicine, School of Medicine, Stanford University, Stanford, CA, United States; ^2^ Division of Gastroenterology, Hepatology, and Nutrition, Department of Pediatrics, School of Medicine, Stanford University, Stanford, CA, United States; ^3^ Department of Internal Medicine, Liver Research Institute and Seoul National University College of Medicine, Seoul, Republic of Korea; ^4^ GI Drug Discovery, Takeda Pharmaceuticals, Lexington, MA, United States; ^5^ Global Computation Biology, Takeda Pharmaceuticals, San Diego, CA, United States; ^6^ Institute for Immunity, Transplantation and Infection, School of Medicine, Stanford University, Stanford, CA, United States

**Keywords:** GPR15, GPR15L, colon cancer, T cells, tumor-microenvironment

## Abstract

**Introduction:**

The chemoattractant receptor, G protein-coupled receptor 15 (GPR15), promotes colon homing of T cells in health and colitis. GPR15 function in colon cancer is largely unexplored, motivating our current studies.

**Methods:**

In human study, immune cells were isolated from tumor tissues and healthy surgical tumor margins (STM), and their proportions as well as expression of GPR15 was analyzed by flow cytometry. In mouse studies, colon cancer was induced in GPR15-deficient (KO) and GPR15-suficient (Het) mice using azoxymethane (AOM) and dextran sulfate sodium (DSS) solution in drinking water. Serial endoscopy was performed in mice to monitor and visualize the distal region of colon. Mice were euthanized 10 weeks after the initial DSS administration, and the colon length and the number of polyps were recorded. Next, we identified the effects of GPR15L on established tumors in the MC38-colorectal cancer (CRC) mouse model. Immune cells were isolated from the mice colons or tumors and assessed by flow cytometry.

**Results:**

Our analysis of human CRC tissue revealed a significant reduction in GPR15^+^ immune cell frequencies in tumors compared to ‘tumor-free’ surgical margins. Similarly, our data analysis using The Cancer Genome Atlas (TCGA) indicated that lower GPR15 expression is associated with poor survival in human colon cancer. In the AOM/DSS colitis-associated colon cancer model, we observed increased colonic polyps and lower survival in *Gpr15* ^+^-KO compared to *Gpr15*-Het mice. Analysis of immune cell infiltrates in the colonic polyps showed significantly decreased CD8^+^ T cells and increased IL-17^+^ CD4^+^ and IL-17^+^ CD8^+^ T cells in Gpr15-KO than in Het mice. Consistent with a protective role of GPR15, administration of GPR15L to established tumors in the MC38-CRC model increased CD45^+^ cell infiltration, enhanced TNFa expression on CD4+ and CD8+ T cells at the tumor site and dramatically reduced tumor burden.

**Discussion:**

Our findings highlight an important, unidentified role of the GPR15-GPR15L axis in promoting a tumor-suppressive immune microenvironment and unveils a novel, colon-specific therapeutic target for CRC.

## Introduction

CRC is a complex, heterogenous disease and is a leading cause of cancer-related deaths world-wide (1). CRC is associated with a multitude of risk factors such as obesity, low-fiber and high-fat diet, heavy alcohol consumption, smoking, family history, inflammatory bowel disease (IBD) and so on (2–4). Patients diagnosed with chronic colitis, such as Crohn’s disease (CD) and ulcerative colitis (UC), are prone to developing a distinct type of CRC, termed colitis-associated colon cancer (CAC) (5–7). Chronic, relapsing inflammation results in severe intestinal damage and sets the stage for colon cancer development by shaping a pro-tumor immune microenvironment. The intricate balance between tumor infiltrating immune cells including effector cells such as T cells, NK cells, immune-suppressive regulatory T cells (Tregs), myeloid cells, dendritic cells, B cells and their interactions with cellular and molecular components in the tumor microenvironment (TME) influence tumor development and clinical outcomes. For instance, a prominent Th1/Th2 phenotype shift, expansion of myeloid-derived suppressor cells (MDSC), tumor-associated macrophages (TAMs), decreased CD8^+^ T cells, along with increased representation of certain inflammatory mediators such as IL-6, IL-17 contribute to tumor advancement. Conversely, infiltration of tumor-fighting cytotoxic CD8^+^ T cells, Th1 cells and generation of mediators such as IFNγ in the TME have been shown to collaborate in mounting anti-tumor responses resulting in cancer regression (8). Several studies highlight a positive correlation between increased infiltration and high densities of CD3^+^ T cells (effector CD4^+^ or CD8^+^ T cells) at tumor sites to disease-free survival in CRC patients (9–12). Interaction between chemokine and chemokine receptors facilitate traffic of T cells to the tumor. Pre-clinical development of modulators of chemokines and chemokine receptors is being actively pursued to be used as stand-alone or combination therapy for various oncology indications including CRC (13).

GPR15 (also known as BOB) is seven-transmembrane domain, class A G-protein coupled receptor (GPCR), first identified as a co-receptor for human immunodeficiency virus (HIV) or simian immunodeficiency virus (SIV) (14–16). GPR15 is expressed on regulatory and effector CD4^+^ T cells, fetal thymic epidermal T cells, B cells and plasmablasts. Studies from our group and others have identified GPR15 as a mucosal chemoattractant/trafficking receptor that facilitates migration of effector and regulatory T cells to sites of inflammation in the large intestine and plays a crucial role in colitis pathogenesis (17). GPR15 was deorphanized with the identification of its cognate chemoattractant ligand, named GPR15L, encoded by the human gene *C10orf99* and mouse *2610528A11Rik*. GPR15L is expressed in human and mouse epithelial cells exposed to the environment, in organs such as the colon and skin (18, 19). In contrast to the well-studied role of GPR15 in colitis and other inflammation conditions, the function of GPR15-GPR15L signaling axis in CRC is poorly understood. In particular, experimental evidence linking immune functions of GPR15 and GPR15-mediated mechanisms to CRC development is rudimentary.

In this report, we delineate a novel tumor-suppressive function of GPR15 using CRC mouse models and patient samples. We demonstrate using human colon cancer samples that GPR15 expression and GPR15^+^ T cells are significantly reduced in tumors compared to surgical tumor margins (STM), which are regarded ‘tumor-free’. In an AOM-DSS mouse model of CAC, *GPR15* deficiency resulted in increased tumor burden, morbidity, and mortality concomitant with reduced T cell infiltration (especially CD8^+^ T cells) in the tumor microenvironment. Moreover, the immune infiltrates in the colon polyps of *Gpr15-*KO mice as well as the human CRC tumor samples with reduced GPR15 expression showed significant increases in the population of pro-inflammatory immune cells such as IL-17^+^ CD4^+^ and IL-17^+^ CD8^+^ T cells. Consistent with a protective role of GPR15, administration of GPR15L to established tumors in the MC38-CRC mouse model resulted in a significant reduction in tumor burden and increased survival. The experimental evidence from mouse colon cancer models and human disease strongly supports GPR15 function in immune cell infiltration of colon polyps/tumors to alter the ‘local’ immune contexture to prevent tumor development and/or mediate tumor resolution.

## Materials and methods

### Patient samples

Colon resection samples were obtained from patients undergoing adenocarcinoma indicated colectomies with approval by the Stanford University Institutional Review Board and after obtaining informed patient consent. The clinicopathological features of the patient samples are summarized in **Table S1**. After removal of the serosa, mesenteric fat and muscularis externa, leukocytes were isolated from the lamina propria as described (20). Cells were resuspended in RPMI containing 5% FBS and re-stimulated with 50 ng/ml phorbol 12-myristate 13-acetate (PMA, Sigma-Aldrich) plus 1 μg/ml ionomycin (Sigma-Aldrich) in the presence of 5 μg/ml Brefeldin A (BioLegend) for 4 hours at 37°C prior to staining for flow cytometry analysis.

### Analysis of human COAD TCGA datasets

GPR15 and GPR15L expression in human COAD TCGA cohorts was analyzed using TIMER2.0 (http://timer.cistrome.org) and GEPIA (http://gepia.cancer-pku.cn) webservers (21, 22). Briefly, TIMER2.0 ‘Gene_DE’ module was used to assess differential expression of GPR15 and GPR15L in tumor and adjacent normal tissues in TCGA cancer types collected from GDAC firehose website. TIMER2.0 extracts raw counts and Transcripts Per Million (TPM) from RSEM results. Additional analysis of GPR15 and GPR15L expression in human COAD tumor tissue and normal tissue as well as its association with patient survival was done using GEPIA webserver which utilizes RNAseq data from TCGA and GTEx projects (source: UCSC Xena project; http://xena.ucsc.edu).

### Analysis of GPR15 and GPR15L expression using single cell RNA-sequencing data from colorectal tumors

Single cell RNA-Seq data published in Lee et al. (23) was downloaded from ArrayExpress repository (E-MTAB-8410). Analysis was performed on preprocessed and aggregated single cell expression data of border regions of 9 colorectal tumors as well as adjacent non-malignant colon tissue. Gene expression matrices were log-normalized and scaled. About 2,000 variable genes, identified using the vst method, were subjected to principal component analysis (PCA). The number of principal components (PCs) used for nonlinear dimensional reduction analysis (t-SNE and UMAP) was chosen according to the PCElbowPlot function and JackStrawPlot function. For cell clustering, FindClusters method was parameterized with different resolutions, while for cell annotation we utilized the ontology-based inferred labels from Lee et al. (23).

### *In vivo* studies

C57BL/6 and B6.SJL mouse strains were purchased from Jackson Laboratory and bred in-house. GPR15-GFP mice, generated as described (17), were backcrossed 10 times onto the C57BL/6 background before use in experiments. *Gpr15*-Het (*Gpr15^gfp/+^*) and *Gpr15*-KO (*Gpr15^gfp/gfp^*) mice used in our studies are ‘knock-in’ mice in which the endogenous *Gpr15* is replaced with the sequence for *GFP*. Animals were maintained in accordance with the US National Institutes of Health guidelines, and experiments were approved by Stanford University Institutional Animal Care and Use Committee.

To induce colon tumors using AOM-DSS, 8-week old mice were injected intraperitoneally with the procarcinogen azoxymethane (AOM) (10 mg/kg of body weight, Sigma-Aldrich). After the first week, the mice received drinking water supplemented with 2% DSS (MP Biomedicals; MW, 36-50 kDa) for 7 days, followed by 2 weeks of regular water. The DSS administration was repeated twice by providing mice with 2% DSS in drinking water (24–26). Body weight was measured once a week for 10 weeks, during the course of the AOM-DSS treatment. Serial endoscopy was performed at day 24, 45 and 66 in live mice to visualize the distal region of the colon and monitor tumor growth. Mice were euthanized 10 weeks after the AOM administration, and the colon length and the number of polyps were recorded. Immune cells were isolated from the mice colons and assessed by flow cytometry.

The murine colon adenocarcinoma cell line, MC38, was kindly provided by Jeanette Baker, Stanford University, USA and maintained in DMEM supplemented with 10% FBS, 1mM L-Glutamine, Penicillin (100 µg/ml), Streptomycin (100μg/ml) and 2-Mercaptoethanol (50 μM), at 37°C in a 5% CO_2_ incubator. For tumor induction, MC38 cells (5 × 10^5^ cells/site) were implanted subcutaneously (s.c.) in the left and right flank of female *GPR15*-Het or *GPR15*-KO mice. When the tumor diameter reached 4-5 mm (on day 4), mice were given intratumoral (i.t.) injection of 50 μl of PBS (left flank) or murine recombinant GPR15L procured from PeproTech (right flank, 2.5 μg in PBS/injection), 3 times per week for 2 weeks. Tumor size was measured 3 times per week using a digital caliper and mice were euthanized when tumors reached a size of 1.7 cm in each dimension. Immune cells were isolated from tumor tissues using the cell isolation methods described above and stained for flow cytometry analysis.

### Mouse colonoscopy

Tumor burden and the size of tumors (referred to as polyps in the results and figures) in the distal part of the colon were determined by micro-colonoscopy endoscopy at day 24, day 45 and day 66 of AOM-DSS treatment as described (27). Mice were anesthetized using 2% isoflurane (Forane, Baxter, Deerfield, Il). The colorectal lumen of the sedated animals was cleaned with phosphate-buffered saline (PBS) pre-warmed to 37°C and fecal pellets removed. The endoscope (G110074, Pentax) was then inserted slowly into the anus of the animals. The video processing, light, and airflow were delivered by the video processor (EPK-1000, Pentax). The white-light endoscopy was recorded using GrabBee software on a laptop connected using a VGA to HDMI connector.

### Cell isolation

Single cell suspension of leukocytes was prepared from mouse spleen, Peyer’s patches, mesenteric and peripheral (including inguinal, brachial sciatic and axillary) lymph nodes by mechanically dispersing the tissues through a stainless steel 100 μm wire mesh into Hank’s buffered salt solution (HBSS; Mediatech, Inc.) containing 2% BCS. Splenic red blood cells were lysed with Red Blood Cell Lysis buffer (Sigma-Aldrich). Intraepithelial and lamina propria lymphocytes were isolated from the small intestine and colon as described previously (28). After excision of the Peyer’s patches, the intestines were cut into approximately 0.5 cm pieces and cleansed with HEPES-buffered RPMI medium containing 5% BCS. To isolate lamina propria (LP) lymphocytes, the tissue pieces were incubated in 2mM EDTA for 15 minutes (x 2 rounds) followed by 3 rounds of collagenase (0.7 mg/ml) digestion at 37°C (20 minutes each), and the supernatant was collected, pooled, and washed. Centrifugation in a 40% and 70% Percoll step-gradient produced enriched colon lamina propria lymphocytes at the interface, which were collected, washed, and stained for flow cytometry analysis.

### Flow cytometry and antibodies

For intracellular cytokine assays, cells were re-stimulated with PMA (50 ng/ml) and ionomycin (1 μg/ml) in the presence of Brefeldin A (5 μg/ml) for 4 hours at 37°C, prior to flow cytometry staining. Dead cells were first excluded using Zombie Aqua™ Fixable Viability Kit (BioLegend). Cells were then incubated with fluorescence conjugated antibodies in HBSS containing 2% BCS at 4°C for 30 minutes to stain surface markers. Surface-stained cells were permeabilized with Cytofix/Cytoperm kit (BD Biosciences) and incubated with fluorescent-labeled antibodies to different cytokines in HBSS containing 2% BCS at 4°C for 30 minutes. The following antibodies were used for flow cytometry in the mouse studies: CCR1 (S15040E), CCR9 (CW-1.2), CD3 (17A2, 145-2C11), CD4 (RM4-5, GK1.5), CD11b (M1/70), CD11c (N418), CD19 (1D3/CD19), CD25 (PC61), CD45 (30-F11), CD103 (2E7), CD117 (ACK2), CD206 (C068C2), F4/80 (BM8), GPR15 (S150421), Gr-1 (RB6-8C5), IL-4 (11B11), IL10 (JES5-16E2), IL-22 (Poly5164), MHC-II(I-A/I-E) (M5/114.15.2), NK1.1 (PK135), TGF-b (TW7-16B4), TNF-a (MP6-XT22) from BioLegend; CD8 (53-6.7), CD45 (30-F11), IFN-g (XMG1.2), IL10 (JES5-16E2), IL-12(p40/p70) (C15.6), IL-17A (TC11-18H10) from BD Biosciences; Foxp3 (IC8214A) from R&D Systems. For staining cells from human colon tissues, the following antibodies were used: CCR1 (5F10B29), CD3 (UCHT1), CD8 (SK1, RPA-T8), CD11c (3.9), CD19 (HIB19), CD25 (BC96), CD45RO (UCHL1), CD68 (Y1/82A), CD117 (104D2), CD127 (A019D5), CD206 (15-2), GPR15 (SA302A10), IL-10 (JES3-19F1), IL-17A (BL168), IFN-g (B27, 4S.B3), TNF-a (Mab11), Perforin (B-D48), mouse IgG2a isotype control (MOPC-173) from BioLegend; CD4 (RPA-T4), CD206 (19.2) from BD Biosciences; GPR15 (FAB3654P) from R&D Systems. Data was acquired on an LSRII or Fortessa cytometer (BD Biosciences) and analyzed with FlowJo software (FlowJo, LLC). Cytokine secretion was calculated by subtracting the signal from unstimulated cells from that of stimulated cells.

### Histology of colon tissues

Hematoxylin and Eosin (H&E) staining was performed by the Histology Service Center (HIC) at Stanford University. Histological scoring was performed in a blinded fashion by two independent observers under the guidance of the scoring method as described elsewhere (26). The histology score was calculated using the following formula:


$$\begin{array}{c} \text{Histology score}=(\text{Grade of Inflammation}+\text{Grade of extent}+\text{Grade of crypt damage})\\ \times \text{Grade of percent involvement}. \end{array}$$

### Statistical analysis

Student’s *t-test*, Mann–Whitney U test, Mantel-Cox test or Two-way ANOVA was performed using Prism (GraphPad) to analyze all experimental data, unless otherwise stated. A p value of less than 0.05 was considered statistically significant.

## Results

### Reduced GPR15 expressing immune cells in human colon cancer

**Figure 1:**
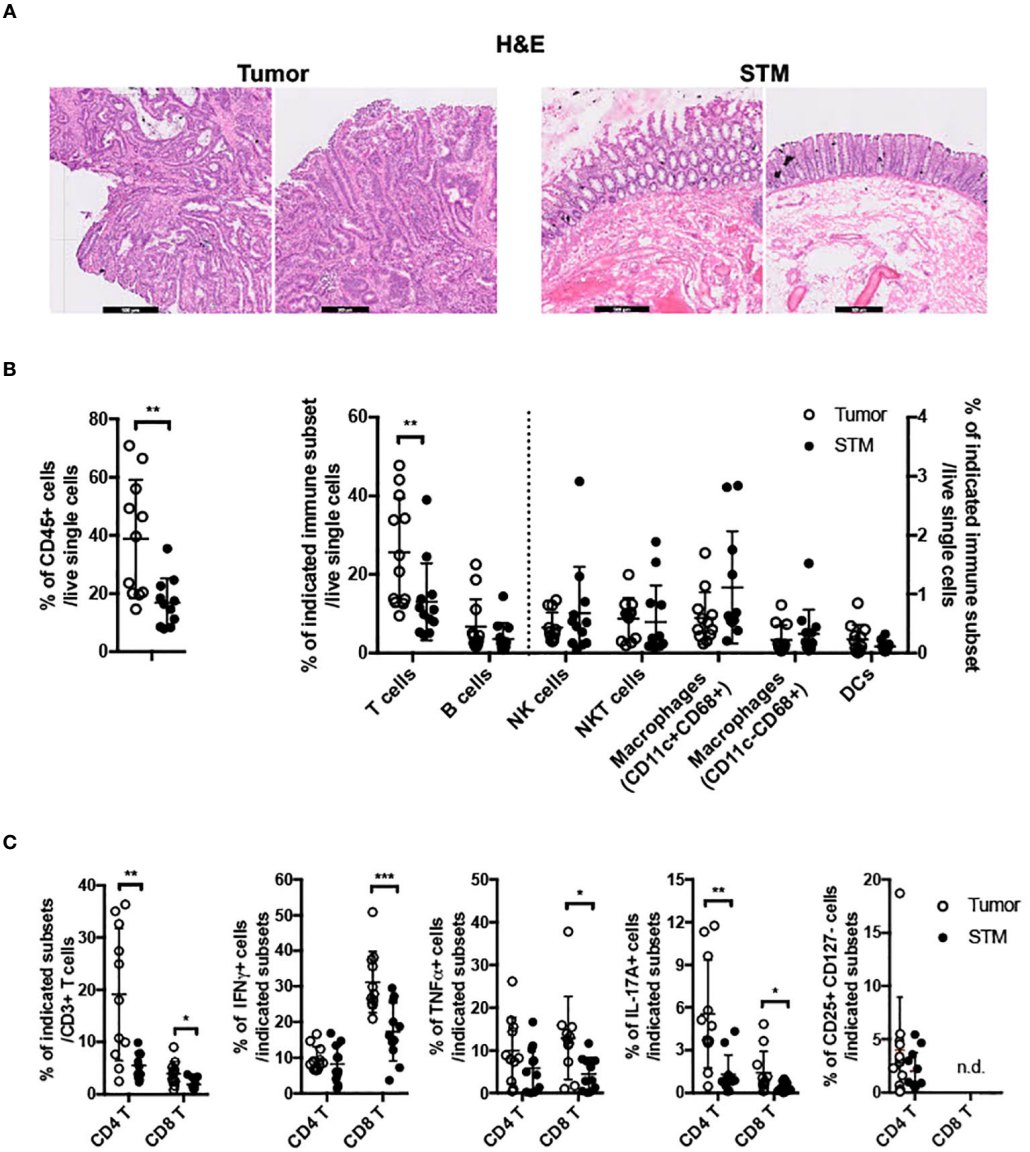
Frequencies of immune cells in human colon cancer. **(A)** H&E staining of tissue sections from human CRC, tumor and STM. Scale bar, 500μM. **(B)** Flow cytometry analysis of total CD45^+^ immune cells (left) and innate and adaptive immune cells (right) in tumor and STM. **(C)** Flow cytometry analysis of the frequencies of total and cytokine (IFNγ, TNFα, and IL-17A) secreting CD4^+^ and CD8^+^ T cells and CD4^+^ regulatory T cells (CD4^+^ CD25^+^ CD127^-^) in tumor and STM. n=10-11. Students *t-test*. *p<0.05, **p<0.01, ***p<0.001.

To gain insights into GPR15-mediated immune function(s) in colon cancer, we assessed tumor-associated immune environment changes and the frequency of GPR15^+^ immune cells in the tumor compared to surgical tumor margin (STM) of human CRC samples using flow cytometry. STMs were tumor-free as evidenced by H&E as well as clinical pathologist’s assessment and used *in lieu* of healthy controls (**Figure 1A**). The core gating strategy for the FACS analysis is outlined in **Supplementary Figure 1**. Our analysis revealed increased CD45^+^ immune cells (~2-3 fold) and among CD45^+^ cells, a significant increase only in T cells but not in B cells, NK cells, mast cells, or macrophages in the tumor samples compared to STM (**Figure 1B**). Moreover, the frequencies of CD4^+^ and CD8^+^ T cells were significantly up-regulated in the tumor tissues, with substantial increases in IFNγ, TNFα and IL-17A expression in CD8^+^ T cells and augmented IL-17A expression in CD4^+^ T cells (**Figure 1C**). Additional immune features of note included a striking increase in the CD4/CD8 T cell ratio in the tumor samples compared to STM and marked changes in the relative abundance of effector T cell subsets. A comparative analysis revealed a notable shift in the ratio of IFNγ^+^ CD8^+^ to IL-17A^+^ CD8^+^ T cell frequencies (Tumor- 31.2% versus 1.4%; STM- 17.3% versus 0.4%). The ratios of IFNγ^+^ CD8^+^ to TNFα^+^ CD8^+^ T cells also showed alteration favoring more TNFα^+^ cells while the ratios of TNFα^+^ CD8^+^ to IL-17A^+^ CD8^+^ T cells showed no change. A similar trend was observed in the relative frequencies of IFNγ^+^ CD4^+^ to IL-17A^+^ CD4^+^ T cells (Tumor 9.7% versus 5.5%; STM 8.2% versus 1.3%). Interestingly, the ratio of TNFα^+^ CD4^+^ to IL-17A^+^ CD4^+^ T cells was also skewed towards more IL-17A^+^ CD4^+^ T cells in the tumor tissues versus STM while the ratios of IFNγ^+^ CD4^+^ and TNF^+^ CD4^+^ T cells showed a small change towards more TNFα. Consistent with a previous report (29), we noticed an increase in regulatory CD4^+^ T cells in the tumor samples compared to STM though it did not reach significance. The change in the frequencies of tumor-infiltrating immune subsets observed in our human CRC cohort is reminiscent of tumor immune environments with abundant immunosuppressive effector T cells.

**Figure 2:**
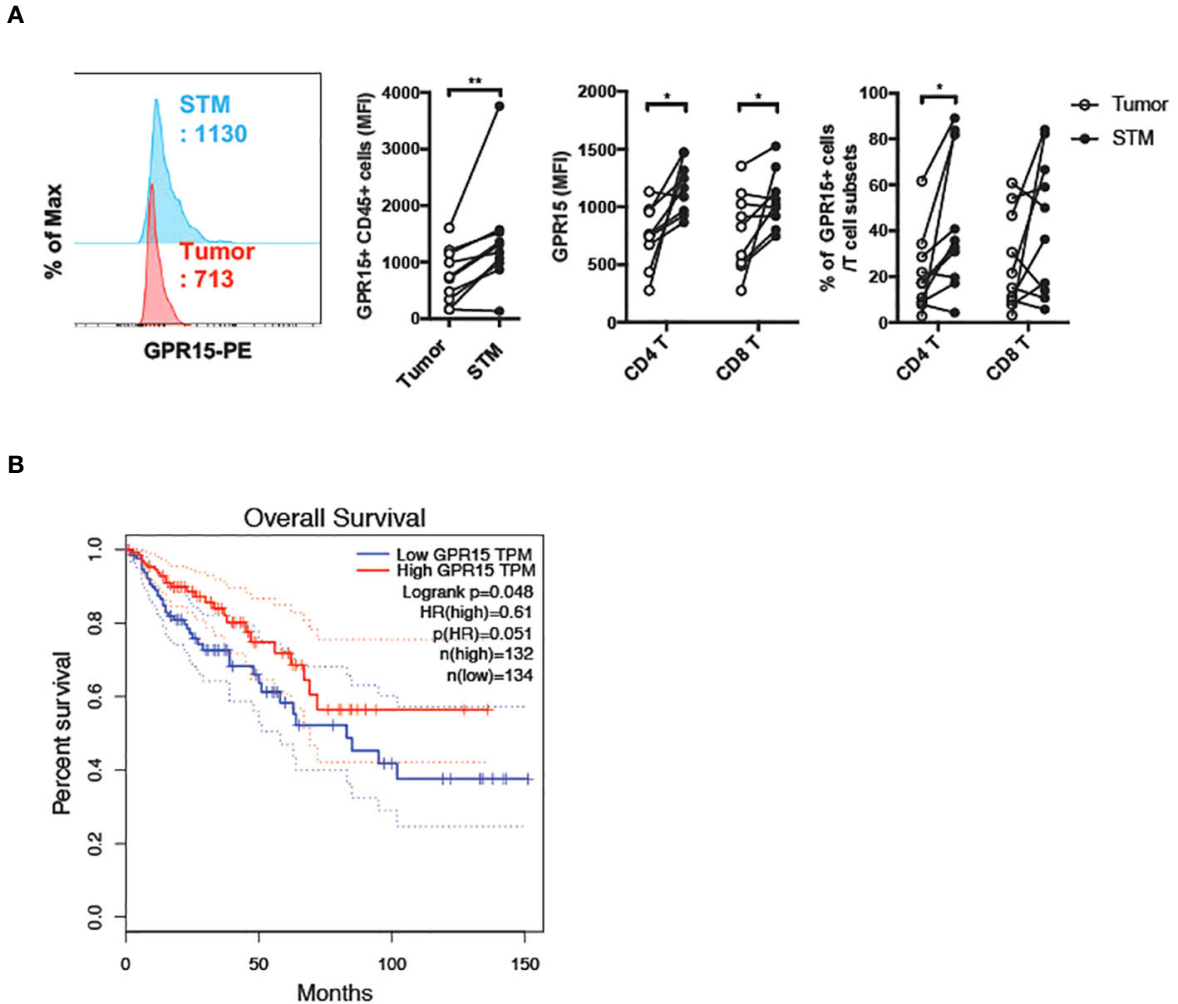
Reduced GPR15 expressing immune cells in human colon cancer. **(A)** GPR15 expression on immune cells isolated from CRC tissue samples, tumor and STM was analyzed by flow cytometry. Data shown are mean fluorescence intensity (MFI) values for GPR15 and frequency of GPR15^+^ cells in CD45^+^ cells (left), CD4^+^, and CD8^+^ T cells (right). n=10-11. Students *t-test*. *p<0.05, **p<0.01. **(B)** Expression of *GPR15* mRNA and its correlation to patient survival in human colon adenocarcinoma (COAD).

To assess if there was a correlation between GPR15 expression and altered immune cell frequencies in our CRC cohort, we examined GPR15 expression in the immune infiltrates from the same human CRC tissues. Our results indicate that both GPR15 expression (reported as mean fluorescence intensity; MFI) and the frequency of GPR15^+^ CD45^+^ immune cells was decreased in the tumor tissue compared to STM. Importantly, we noted that GPR15 expression on T cells and frequency of GPR15^+^ CD4^+^ T cells were significantly less in tumors, with a decreasing trend in the frequency of GPR15^+^ CD8^+^ T cells as well (**Figure 2A**). GPR15 expression was also significantly decreased in other immune cell subsets, such as natural killer T cells (NKT), macrophages and B cells (**Supplementary Figure 2**). Our data suggests a potential link between differential GPR15 expression and altered composition of both innate and adaptive immune subsets in human CRC depending on disease status (tumor compared to ‘normal’ tumor-margin) with a marked shift towards certain inflammatory, immune-suppressive effector phenotypes, such as elevated IL-17-expressing CD4^+^ T cells in tumor tissues.

### Lower GPR15 expression is associated with poor survival in human colon cancer

**Figure 3:**
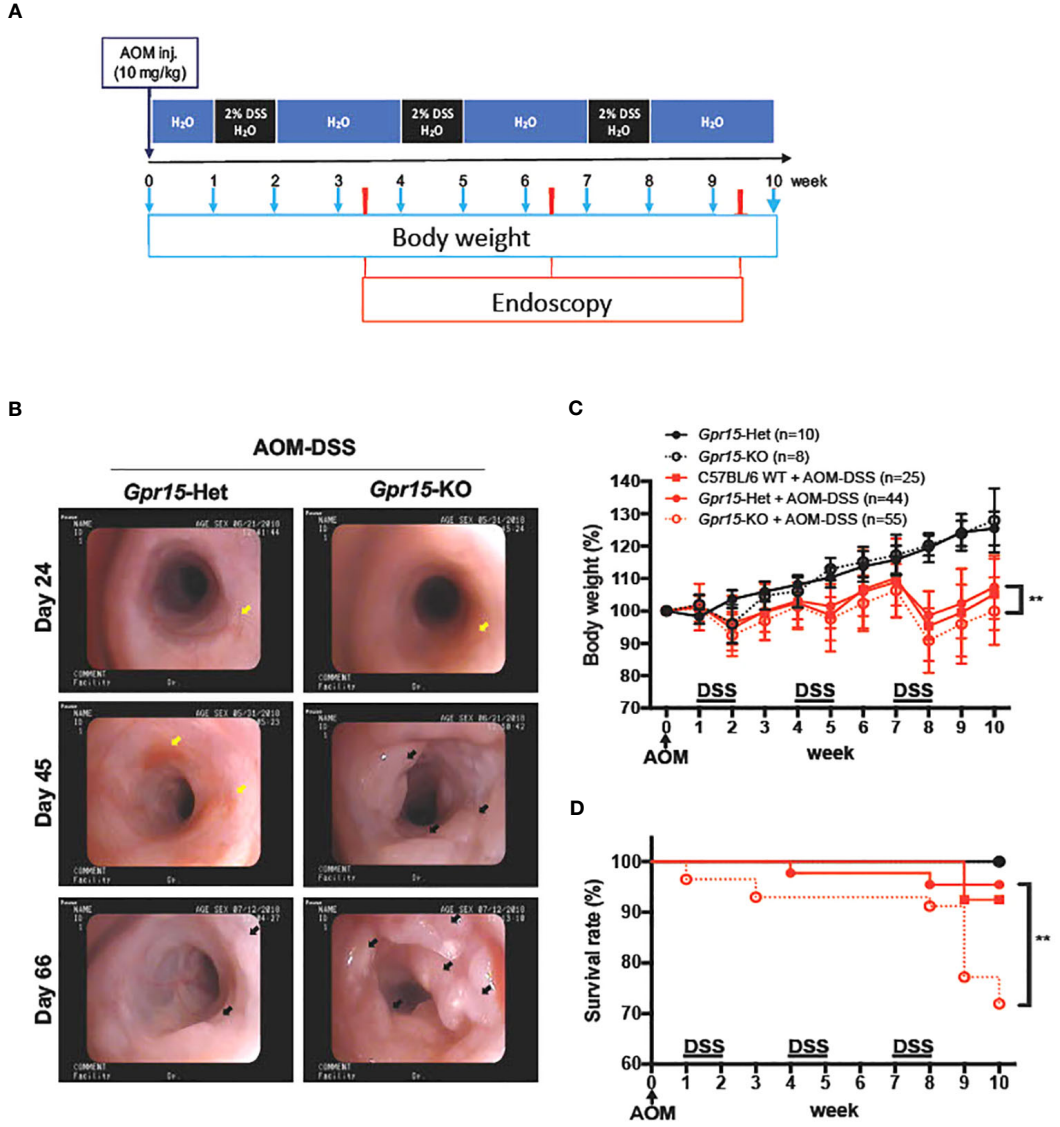
GPR15 deficiency promotes colon tumors and reduces survival in the AOM-DSS murine model of colitis-induced colon cancer. **(A)** Experimental scheme for the AOM-DSS murine colon cancer model with time points for body weight and endoscopy measurements indicated. **(B)** Representative images of the distal colon in *Gpr15*-Het and KO mice obtained by murine endoscopy on days 24, 45, and 66, during the course of AOM-DSS treatment. Inflammation sites are indicated by yellow arrows, and polyps are indicated by black arrows. **(C)** Bodyweight of untreated and AOM-DSS treated wild type, *Gpr15*-Het and KO mice was measured at different time points as shown in A and reported relative to baseline at start of treatment. Mean ± SD is shown. **(D)** Survival rate was assessed during the course of AOM-DSS treatment. Data shown is the sum of 5 independent experiments. **p<0.01 by Mantel-Cox test.

To assess the impact of GPR15 expression and its correlation to patient prognosis in human colon adenocarcinoma (COAD), we analyzed The Cancer Genome Atlas (TCGA) transcriptomics data using two different publicly available analysis platforms, GEPIA (21) and TIMER2.0 (22). Our analysis revealed significantly reduced GPR15 mRNA expression in tumors compared to tumor adjacent normal tissue in COAD. Importantly, low GPR15 expression dramatically reduced/shortened patient survival, suggesting that decreased GPR15 expression may be linked to colon cancer disease progression (**Figure 2B**; **Supplementary Figure 3A** (top panel), **Figure 3B** (left panel)). GPR15L levels were also significantly lower in tumors compared to normal tissue in human COAD though it did not significantly affect patient survival (**Supplementary Figure 3A** (bottom panel), and **Figure 3B**; middle and right panels). As a complementary, unbiased approach to analyze GPR15 expression in human CRC, we queried a publicly available human CRC single cell RNA-seq (scRNA-seq) data set (23). Our analysis revealed that GPR15 expression was confined to immune cells (**Supplementary Figures 4A-C**) while GPR15L was expressed in non-immune, epithelial cells (**Supplementary Figures 4A**, **5A, B**). Among immune cells, GPR15 expression was particularly evident in CD8^+^ alpha-beta T cells, gamma-delta T cells, Th17 cells, plasma cells, IgA^+^ plasma cells and CD4^+^ T cells (**Supplementary Figure 4B**). In agreement with observations in our patient cohort, we observed reduced GPR15 as well as GPR15L expression in the tumor compared to tumor border and normal tissue adjacent to tumor border (**Supplementary Figures 4C**, **5A**). Reduced GPR15 expression on tumor-infiltrating T cells in human colon cancer and its impact on disease pathogenesis has not been previously reported and prompted us to investigate its significance using mouse colon cancer models.

### GPR15 deficiency promotes colon tumors and reduces survival in the AOM-DSS murine model of colitis induced colon cancer

**Figure 4:**
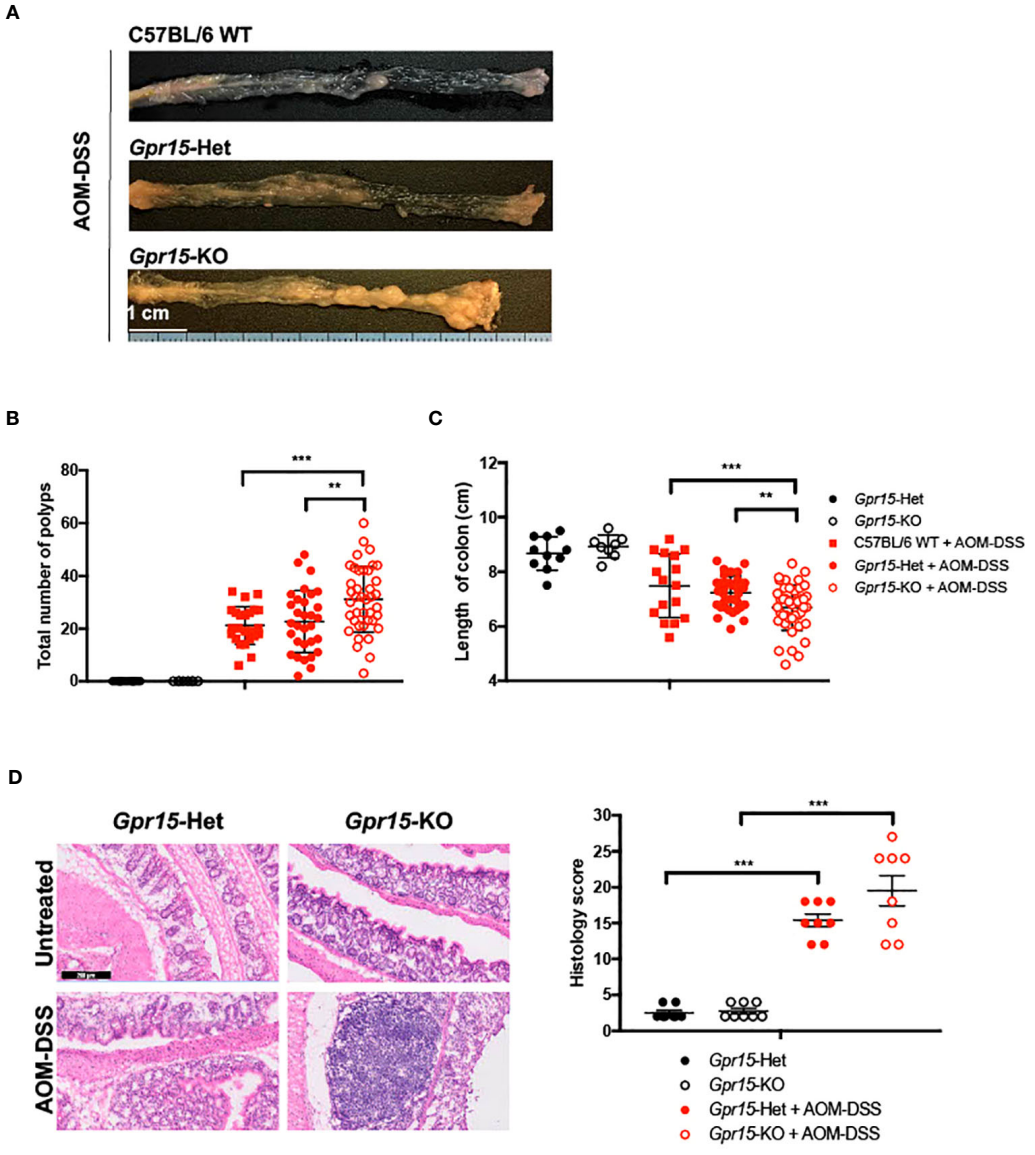
GPR15 deficiency increases the incidence of colon tumors and disease severity in the AOM-DSS murine model of colitis-induced colon cancer. **(A)** Representative pictures of the mouse colons, **(B)** the total number of polyps and **(C)** colon length after the completion of 10-weeks of AOM-DSS treatment. Data shown is the sum of 5 independent experiments. **p<0.01, ***p<0.001 by Students *t-test*. **(D)** Representative H&E staining (left) and histological evaluation of disease severity (right) in the colon sections obtained from *Gpr15-*Het or *Gpr15-*KO mice with or without AOM-DSS treatment (n=8 per group). Scale bar, 200μm. Mean ± SD; ***p<0.001 by Mann Whitney U-test.

To examine the role of GPR15 in CRC development, we used the well-characterized AOM-DSS induced colitis-associated colon cancer (CAC) experimental model to study the course of tumor formation and development in *Gpr15*-sufficient (Het) and *Gpr15*-deficient (knockout; KO) mice (17) (**Figure 3A**). The azoxymethane-dextran sulfate sodium (AOM-DSS) colon carcinogenesis model recapitulates the multi-step, tumor development sequence and histopathological features of human CRC and is valuable to examine the role of inflammation in colon cancer development. In this model, the colon-tropic carcinogen AOM is used in combination with the inflammation-causing agent DSS, which erodes the colon epithelia resulting in severe chronic colitis characterized by bloody diarrhea and tumor formation in the middle and distal colon of murine within 10 weeks (24, 30–32). Based on our observation that GPR15^+^ T cells are reduced in the tumor microenvironment in human CRC, we posited that GPR15 deficiency would result in enhanced disease susceptibility and severity in part by influencing T cell infiltration to the colon in the AOM-DSS model of CAC. Tumor development was monitored in live mice by endoscopy at week 3, 6 and 9, during the course of AOM-DSS treatment. The polyps induced in the mouse colon by AOM-DSS treatment are considered tumors (33–35) and we refer to the AOM-DSS induced tumors interchangeably as polyps in the reminder of the manuscript. In line with our hypothesis, we observed severe, widespread inflammation in the colon along with increased incidence of colon polyps/tumors in the *Gpr15*-KO group compared to *Gpr15*-Het group in both early and late phases of disease development (Day 24-66) post AOM-DSS administration (**Figure 3B**; **Supplementary Figure 6A**). Importantly, *Gpr15*-KO mice showed significant body weight loss during disease progression (**Figure 3C**), lower survival rate (**Figure 3D**), more colon polyps (of size > 2mm) in the middle and distal colon (measured at week 10; **Figures 4A, B**; **Supplementary Figures 6B, C**), and shorter colon length (measured at week 10; **Figure 4C**) than *Gpr15*-Het mice. Histology analysis and disease severity scoring after AOM-DSS treatment revealed more severe intestinal inflammation in *Gpr15*-KO compared to *Gpr*15-Het mice, as evidenced by increased necrosis of the epithelial layer, crypt damage, and infiltration of leukocytes in the lamina propria (**Figure 4D**). *Gpr15*-Het and *Gpr15*-KO mice that were not treated with AOM-DSS showed no polyp formation and had similar survival rates, while C57BL/6 WT mice treated with AOM-DSS demonstrated similar disease course/severity and survival rates as *Gpr15*-Het mice (**Figures 3C, D**, **4A-C**; **Supplementary Figure 6C**). Our results thus indicate that GPR15 loss led to increased tumor burden, severe disease pathology and poor survival in the AOM-DSS CAC disease model alludes to a protective role of GPR15 in colitis associated colon cancer development.

### GPR15 deficiency alters immune cell phenotypes and function to favor a pro-tumor environment in the AOM-DSS model of CAC

**Figure 5:**
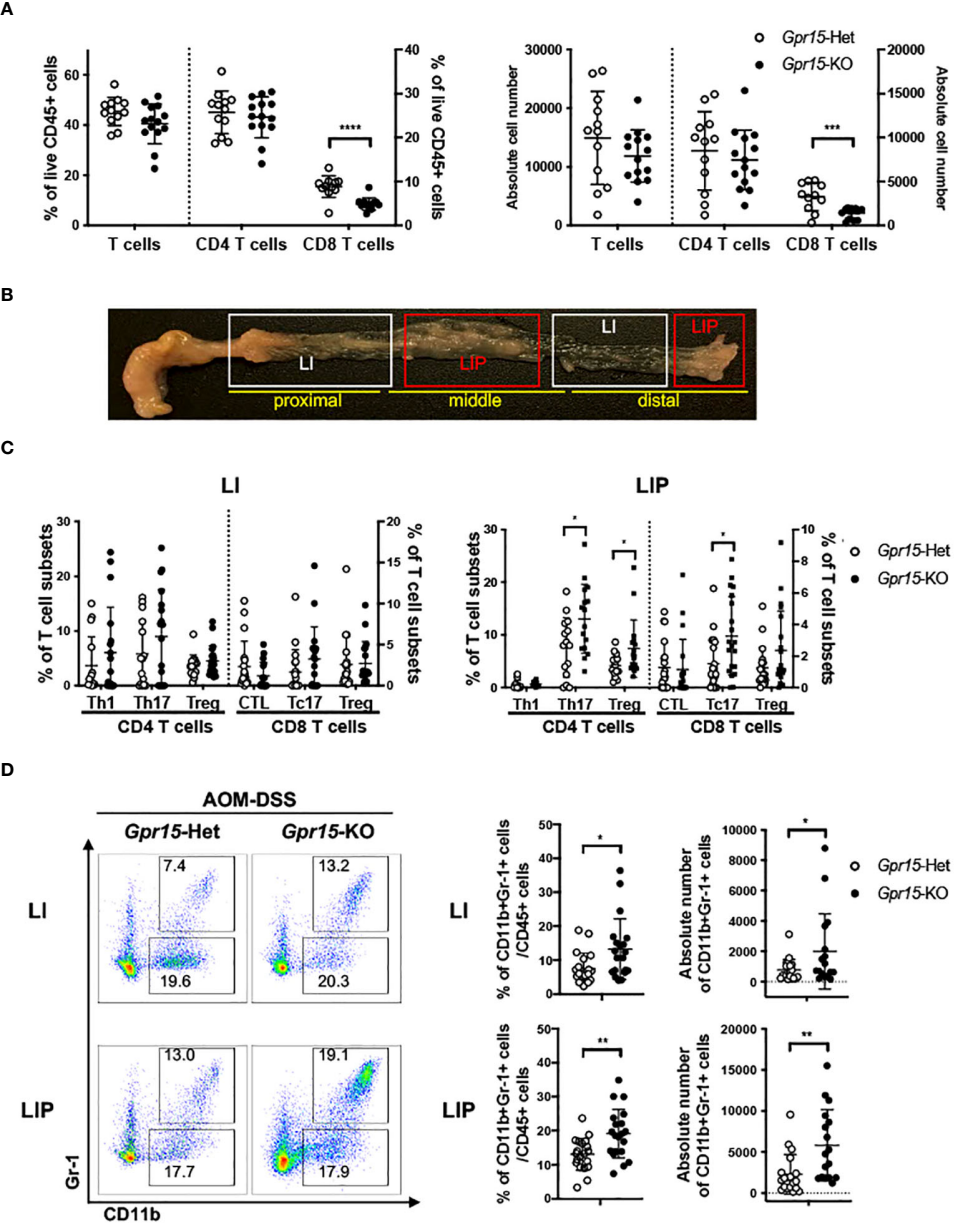
GPR15 deficiency alters immune cell phenotypes and effector T cell populations in the colon tumor microenvironment in the AOM-DSS model. **(A)** Frequencies of CD3^+^ T cells and their subpopulations, CD4^+^ and CD8^+^ T cells, isolated from the colon of AOM-DSS treated *Gpr15*-Het and KO mice as indicated (left). Absolute cell numbers of CD3^+^, CD4^+,^ and CD8^+^ T cells are shown (right). n=12-14. **(B)** Representative picture showing regions of the large intestine without polyps (LI) and with polyps (LIP). **(C)** Frequencies of CD4^+^ and CD8^+^ T cells expressing IFNγ or IL-17A, and Tregs (CD4^+^ CD25^+^ Foxp3^+^) in LI (left) and LIP (right). n=15-20. **(D)** Representative plots (left), frequencies (middle) and absolute cell numbers (right) of MDSCs from LI (upper) and LIP (lower) of *Gpr15-*Het and *Gpr15-*KO mice administered with AOM-DSS. In **(A, C, D)** frequencies and absolute cell numbers are shown as dot plots, mean ± SD. Students *t-test* *p<0.05, **p<0.01, ***p<0.001, ****p<0.0001.

To delineate GPR15-mediated immune mechanisms involved in tumor suppression in the AOM-DSS CAC disease model, we analyzed the immune cells recovered from the colon in *Gpr15*-Het and *Gpr15*-KO mice at week 10, post AOM-DSS treatment by flow cytometry. Our FACS panel included markers to identify innate and adaptive immune subsets including NK cells, myeloid cells, and T cell subsets (**Supplementary Figure 7**). We confirmed that GPR15 expression levels was significantly reduced in different T cell subsets in the *Gpr15*-KO mice compared to *Gpr15*-Het mice (**Supplementary Figure 8**). We observed a decrease in the total number of T cells and a significant reduction in CD8^+^ T cells in *Gpr15*-KO mice compared to *Gpr15*-Het mice (**Figure 5A**). To ascertain tumor-specific changes in the immune environment, we performed a comprehensive assessment of immune cell phenotypes and their functional state in large intestine lesions with polyps/tumors (LIP) and without polyps (LI) of AOM-DSS treated *Gpr15*-Het and *Gpr15*-KO mice (**Figure 5B**). We observed that GPR15 deficiency led to significant increase in frequencies of regulatory CD4^+^ T cells (CD25^+^ Foxp3^+^), IL-17A^+^ CD4^+^ and IL-17A^+^ CD8^+^ T cells in LIP of AOM-DSS treated mice (**Figure 5C**; **Supplementary Figures 9A, B**). Furthermore, we noted a marked increase in the frequency of myeloid-derived suppressor cells (MDSC; CD11b^+^ Gr-1^+^) in the LI and LIP (**Figure 5D**). Interestingly, the colon polyps (LIP) in the AOM-DSS treated *Gpr15*-KO mice showed distinct (e.g., increased MDSCs) as well as shared (e.g., increased IL-17A^+^ CD4^+^ and IL-17A^+^ CD8^+^ T cells) immune features of human CRC with reduced GPR15 expression. The decreased infiltration of CD8^+^ T cells and expanded MDSC population observed in the tumors (LIP) of AOM-DSS treated *Gpr15*-KO mice are common denominators in tumor formation and progression in several cancers including CRC (36). Our further investigation of the immune cell frequencies in the secondary lymphoid organs, such as spleen (SP), peripheral (axillary, brachial, inguinal, and sciatic) lymph nodes (PLN), and mesenteric lymph nodes (MLN) revealed the presence of diverse immune populations at varying frequencies with some notable differences between the tissues of AOM-DSS treated *Gpr15*-Het and *Gpr15*-KO mice (**Supplementary Figure 10**). CD4^+^ and CD8^+^ T cells were significantly decreased in the MLN of AOM-DSS-treated *Gpr15*-KO mice. Our immune analysis in the secondary lymphoid organs thus reveal systemic, tumor-favoring immune signatures in *Gpr15*-KO mice that reflect the changes in the ‘local’ colon tumor immune environment (e.g., increased MDSC, CD4^+^ T cell subsets) and highlight an important role for GPR15-mediated immune mechanisms in colon cancer development. Our findings in the AOM-DSS CAC model lend credence to GPR15 involvement in recruitment of T cells with tumor-suppressive function to the colon and aiding in the evolution of an immune environment unfavorable for tumor growth.

### GPR15 agonism elicits anti-tumor effects in the murine MC38 colon cancer model

**Figure 6:**
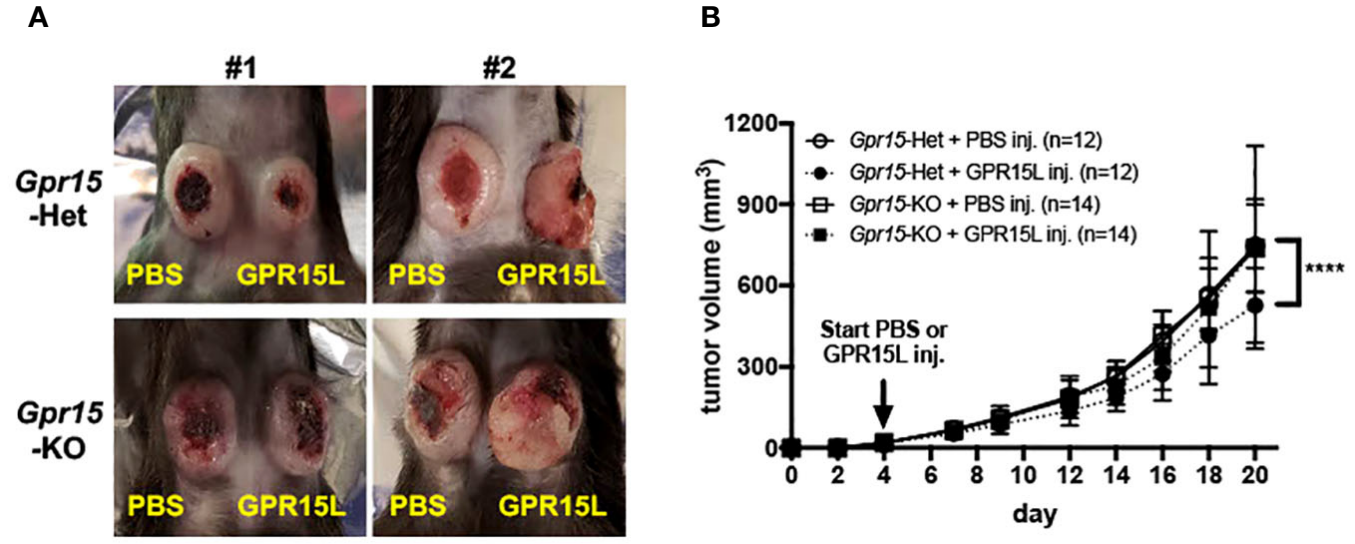
GPR15L treatment facilitates anti-tumor effects in the murine MC38 colon cancer model. **(A)** Representative pictures of PBS (left flank) and GPR15L (right flank) -treated tumors in *Gpr15*-Het (upper) and *Gpr15*-KO mice (lower) at day 21. **(B)** Tumor growth rates in *Gpr15*-Het and *Gpr15*-KO mice with PBS or GPR15L treatment as indicated. Data shown is mean ± SD. Two-way ANOVA ****p<0.0001.

**Figure 7:**
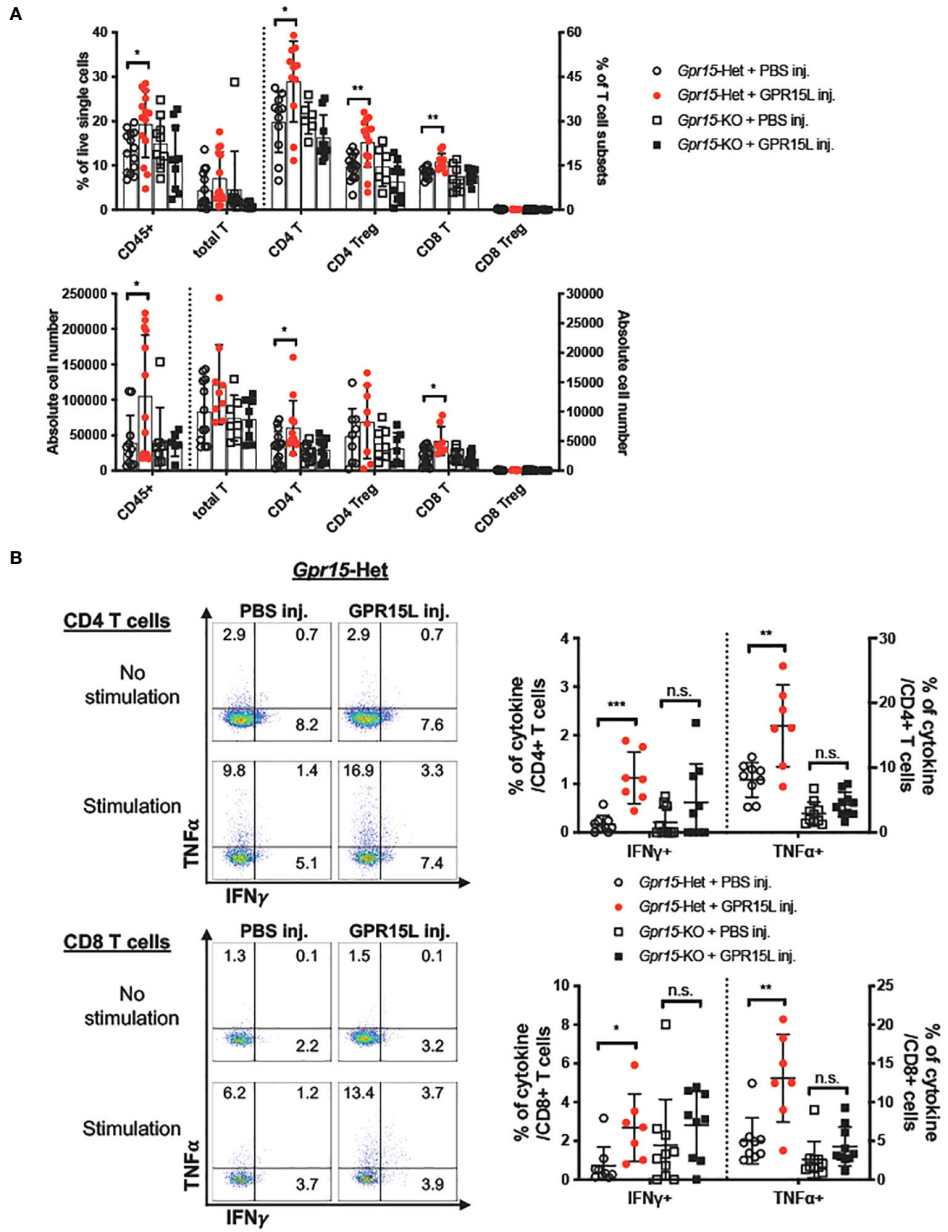
GPR15L treatment facilitates anti-tumor effects by upregulating cytolytic T cells. **(A)** Flow cytometry analysis of CD45^+^ immune cells and T cell subsets in tumors isolated from different treatment groups as indicated. **(B)** Representative flow cytometry plots (left) and frequencies (right) of IFNγ and TNFα-expressing CD4^+^ T (upper panel) or CD8^+^ T cells (lower panel) in tumors of different treatment groups. Data shown in **(A, B)** is mean ± SD. Students *t-test* *p<0.05, **p<0.01, ***p<0.001.

Our findings in human CRC and murine AOM-DSS colon carcinogenesis model highlight a novel function of GPR15 as a tumor-suppressor by modulating the immune microenvironment in colon cancer. To further delineate the role and explore the translational relevance of our findings, we examined if GPR15 agonism by GPR15L administration would elicit an anti-tumor effect by influencing immune cell phenotypes and their functionality in the MC38-murine CRC model. The MC38 cell-line was originally derived from a grade-III colon adenocarcinoma that was chemically induced in a female C57BL/6 mouse (37) and is a well-established CRC model for investigating anti-cancer immunity and immunotherapy (38–40). MC38 cells were injected subcutaneously in *Gpr15*-Het and -KO mice, and tumor development was monitored following concomitant intratumoral injection of GPR15L or vehicle (PBS) when tumors reached 4-5 mm in size (Day 4 post-transplant). In line with our hypothesis, GPR15L administration resulted in dramatic shrinkage in tumor size in *Gpr15*-Het but not in *Gpr15*-KO mice (**Figures 6A, B**), affirming a specific function of the GPR15-GPR15L axis in mitigating colon tumor growth. Flow cytometry analysis of GPR15-GPR15L signaling dependent changes in immune cell populations in the tumors (**Supplementary Figure 11**) revealed significant expansion of CD45^+^ T cells, CD4^+^ and CD8^+^ T cells, in particular IFNγ^+^ and TNFα^+^ T cell subsets, in *Gpr15*-Het but not in *Gpr15*-KO mice treated with GPR15L (**Figures 7A, B**). Our results indicate that GPR15-GPR15L axis impairs tumor growth by facilitating the infiltration of ‘cytolytic’ T cells into the tumor microenvironment in the MC38 CRC model. Furthermore, we noticed increased frequency of TNFα^+^ NK cells, IL-12^+^ IFNγ^+^, and TNFα^+^ NKT cells, TNFα^+^ DCs, and macrophages in the GPR15L-injected tumors isolated from *Gpr15*-Het mice (**Supplementary Figure 12**). Whether these changes are a consequence of direct or indirect GPR15 function warrants further investigation. In sum, our studies provide ‘proof-of-concept’ for the therapeutic targeting of GPR15-GPR15L axis in CRC treatment; intratumoral administration of GPR15L attenuates tumor growth in a GPR15-dependent manner by shaping an anti-tumor immune environment enriched in cytolytic T, NK and NKT cells.

## Discussion

We previously identified an important function of GPR15 in T cell traffic to the inflamed colon and colitis pathogenesis (41, 42). In the present study, we describe a first and novel function of GPR15 in colon cancer: GPR15 plays a crucial role in the colon recruitment of immune cells and shaping of the colon immune microenvironment to mediate a protective, tumor-mitigating effect. This is demonstrated by our *in vivo* studies in the AOM-DSS murine model of CAC, where GPR15 deficiency resulted in a significant reduction of T cells with tumor-suppressive function in the colon, such as effector CD8^+^ T cells and the ability of GPR15L administration to shrink established MC38 tumors in *GPR15*-sufficient but not in *GPR15*-deficient mice. In support of an anti-tumor function of GPR15 in human CRC, GPR15 expression as well as GPR15^+^ immune cells were significantly reduced in human colon tumors and lower GPR15 expression correlated with decreased survival of COAD patients. Our studies offer a strong rationale for modulation of GPR15-GPR15L axis as a colon-specific approach to treat CRC by enhancing effector immune cell responses culminating in tumor rejection.

Our analysis of immune infiltrates in human colon cancer and AOM-DSS mouse CAC model indicated that GPR15 expression may modulate the relative abundance of innate and adaptive immune cell subsets as well as cytokine secreting, effector T cell subsets in the colon tumor microenvironment (TME) in favor of tumor regression. In our human CRC samples, T cells were the predominant population in tumor tissues compared to STM among immune infiltrates analyzed and among T cells, a higher frequency of CD4^+^ T cells compared to CD8^+^ T cells was noted (19.1% vs 3.9%). The ‘T cell rich’ TME in our patient cohort shows consensus with previous reports as well as studies where higher ratios of CD4/CD8 T cells have been reported in human colon cancer samples (43, 44). Emerging evidence suggests a link between increased T cell infiltration especially of CD3^+^ T cells and favorable patient prognosis. However, the balance between pathogenic versus protective T cells and their interactions in the TME is a critical factor governing clinical outcomes (8, 45). Reduced GPR15 expression and frequency of GPR15^+^ immune cells in the TME correlated with an enrichment of certain inflammatory and immunosuppressive phenotypes in our patient cohort. This was evidenced by significantly increased frequencies of IL-17A secreting CD4^+^ and CD8^+^ T cells, and a trend towards increased CD25^+^ CD127^-^ regulatory CD4^+^ T cells in the tumors compared to uninvolved colon tissue (STM). Interestingly, we also noted a substantial increase in IFNγ<σπ>+</σπ> CD8^+^ T cells in the tumors compared to STM control tissue. Further assessment of frequencies and relative levels of other effector T cell populations in tumor versus STM showed a notable shift in the ratio of IFNγ^+^ to IL-17A^+^ T cell frequencies (on both CD4^+^ and CD8^+^ T cell subsets). The ratio of TNFα^+^ CD4^+^ and IL-17A^+^ CD4^+^ was also skewed towards more IL17A^+^ CD4^+^ in tumor compared with STM. Higher proportion of Th17 cells have been reported in tumor as well as blood of CRC patients and is associated with tumor progression and a poor prognosis (46). Interestingly, Amicarella et al. (47) demonstrated using human CRC samples that tumor infiltration of IL-17^+^ T cells *per se* was not predictive of patient survival. Rather, the spatial location of IL17^+^ T cells within tumors influenced prognosis; intra-epithelial but not stromal Th17 cells was positively correlated with improved survival. Whether GPR15 has a role in spatial distribution of effector cells within tumor regions to establish unique, intratumoral immune niches is currently not known and warrants future investigation.

In the AOM-DSS CAC model, GPR15 deficiency was similarly associated with unique alterations in the composition of immune subsets in the tumor (LIP) and non-tumor (LI) regions of the colon as well as in secondary lymphoid organs. We noted a marked overall decrease in tumor infiltrating CD8^+^ T cells and expansion of myeloid (CD11b^+^ Gr1-1^+^) populations. Of significance was a striking increase in the frequency of IL-17^+^ CD4^+^ (Th17) cells and IL-17^+^ CD8^+^ T cells (Tc17) in the polyps (LIP) but not in LI of *Gpr15-*KO mice. Interestingly, the colon polyps (LIP) in the AOM-DSS treated *Gpr15-*KO mice showed distinct (e.g., expansion of MDSCs) as well as shared (e.g., increased IL-17^+^ CD4^+^ and IL-17^+^ CD8^+^ T cells) immune features of human CRC with reduced GPR15 expression. Although we found no changes in the frequency of CD25^+^ CD127^-^ CD4^+^ T cells (Treg) in tumor versus control tissue in our human CRC data, we observed a significant increase in Treg frequency in the LIP of *Gpr15-*KO mice compared with *Gpr15-*Het mice, reflecting increased tumor burden in AOM-DSS treated *Gpr15-*KO mice. These results differ from some previous findings on GPR15 expression and function in Treg, in UC (48, 49) and CRC (35). However, unlike the studies by Adamczyk et al., that reported a tumor promoting role of GPR15 by focusing primarily on Tregs (47), we have performed a comprehensive analysis of well-described CD45+ immune subsets, including effector T cells, regulatory T cells, NK cells, and myeloid cells, relevant to the colon cancer immune microenvironment. We have tried to monitor and integrate the overall effects from all immune cells that express GPR15. The balance of immune cell subsets in the tumor microenvironment determines disease severity and outcome in colon cancer. We find this to be the case in our human CRC and mouse colon tumor samples, where reduced GPR15 levels (in human tumor samples and in tumors from the AOM-DSS *Gpr15* KO experimental group) is associated with the enrichment of immune subsets with inflammatory and immune-suppressive phenotypes (e.g. IL17^+^ CD4^+^ and CD8^+^ T cells). The enrichment of such inflammatory and immune-suppressive phenotypes in the AOM-DSS *Gpr15* KO group is coincident with increased tumor burden in this group. Our current observations are consistent with recent studies which utilized unbiased, single-cell RNA sequencing approach and demonstrated that GPR15 was expressed in effector T cells and B cells, and not restricted to Tregs in human CRC (23, 49 and **Supplementary Figure 4**). The patient cohort we analyzed was not stratified based on factors such as tumor mutations, IBD history, which could differentially affect the nature and composition of tumor infiltrating immune cells. It is thus possible that the observed differences in immune cell composition could be due to heterogeneity and molecular changes inherent in the human CRC samples and underscore the potential of the TME to influence the infiltration of diverse GPR15^+^ immune cells.

Our results in the MC38 murine colon cancer model demonstrate the potential of GPR15L treatment to alleviate tumor burden in a GPR15-dependent manner. The tumor-suppressing capability of the GPR15-GPR15L axis was associated with a significant influx of CD4^+^ and CD8^+^ T cells into the tumor, in particular IFNγ or TNFα secreting T cells. Increased frequencies of TNFα^+^ NK cells, TNFα^+^ NKT cells and IFNγ^+^ NKT cells were also observed following GPR15L treatment of tumors in *Gpr15*-Het. Anti-tumor effects of NK and Type1 NKT in CRC have been reported (50) and likely contribute to direct or indirect GPR15-GPR15L dependent negative regulation of tumor growth. Unlike the observations in the AOM-DSS model, no alterations in IL-17A^+^ T cell frequencies were observed across treatment groups. Thus, there might be differences in GPR15-dependent infiltration of effector CD4^+^ and CD8^+^ T cells depending on the tumor location and subsequent changes in the TME (subcutaneous versus colon). Furthermore, it is possible that the effects of GPR15L on colon tumor growth may involve both GPR15-dependent and independent mechanisms and warrants further studies. In this respect, it is worth noting that GPR15L has been recently reported to interact with SUSD2 to inhibit colon cancer cell growth via G1 arrest, which is induced by down-regulating cyclin D1 and cyclin-dependent kinase 6 (CDK6) (51). Intriguingly, we noted reduced GPR15 expression on tumor infiltrating CD4^+^ and CD8^+^ T cells in GPR15L-treated *Gpr15*-Het mice compared to the PBS-treated *Gpr15*-Het mice. This could be due to downregulation of surface GPR15 upon GPR15L binding. Indeed, a recent report shows that GPR15L facilitates GPR15 endocytosis using GPR15-expressing cell lines (52). Additional studies are required to understand GPR15L-dependent regulation of surface GPR15 levels on immune cells and its role in GPR15-mediated immune mechanisms in health and inflammatory diseases such as colitis and colon cancer to realize the therapeutic potential of GPR15-GPR15L axis in these indications.

Our current findings on GPR15 expression and function in colon cancer are supported/strengthened by a recent pan-cancer study of GPR15 expression and mutational pattern using data from TCGA and GTEx databases that revealed GPR15 was hypermutated and its expression significantly downregulated in colon adenocarcinoma (53). Importantly, there was a positive correlation between GPR15 expression, CD4^+^ T cell infiltration and patient survival. In the same study, integrated network analysis on GPR15 and GO enrichment analysis revealed potential intersections with proteins involved in mitogenic signaling, cell-cycle regulation and links to immune function in different cancers including colon cancer. The study thus provides a glimpse of possible effects that can be mediated by GPR15 in colon cancer beyond its classical function as a T- cell trafficking protein. Another study on mRNA and miRNA signatures in human COAD patient samples also reported reduced GPR15 expression in tumors and potential miRNA mediated regulation of GPR15 levels in COAD (54). A recent study (55) however, showed that GPR15 is upregulated in human colorectal cancer cells and that silencing of GPR15 inhibited the growth of colorectal cancer cells, using *in vitro* assays. While this suggests that GPR15 may play a differential role in non-immune cells, such as cancer cells, it needs to be tested *in vivo* to understand the impact of GPR15 expression in various cell types in the tumor microenvironment. Of note, our analysis of open-source scRNA-seq data obtained from human colon tumor tissue showed that GPR15 is primarily expressed in immune cells with no detectable expression in non-immune/epithelial cells [**Supplementary Figure 4**; (56)], while the GPR15L was expressed primarily on non-immune (epithelial) cells.

The immune environment in CRC is highly heterogenous and complex, evolves during disease progression and poses significant challenges for development of effective immunotherapies. Prominent among the cellular immune mediators in the anti-tumor response are cytotoxic lymphocytes, in particular (primarily) effector CD8^+^ T cells. They are supported in this endeavor by NK cells, Th1-like type I NKT cells and CD4^+^ IFNγ^+^ Th1 cells. There is a growing appreciation for defects in trafficking of immune cells (e.g., homing deficit of anti-tumor effector T cells) to tumor sites as a key impediment to the success of adoptive T cell transfer-based therapies in cancers including CRC (57). Our current findings that the T cell homing receptor, GPR15 can augment the infiltration of tumor-fighting effector T cells and alter the TME in colon cancer lesions in pre-clinical models hold therapeutic promise in immunotherapy-based tumor eradication using GPR15 agonists and improving patient survival.

## Data availability statement

The original contributions presented in the study are included in the article/**Supplementary Material**. Further inquiries can be directed to the corresponding author.

## Ethics statement

The studies involving humans were approved by Stanford University Institutional Review Board. The studies were conducted in accordance with the local legislation and institutional requirements. Written informed consent for participation in this study was provided by the participants’ legal guardians/next of kin. The animal study was approved by Stanford University Institutional Animal Care and Use Committee. The study was conducted in accordance with the local legislation and institutional requirements. Written informed consent was obtained from the individual(s) for the publication of any potentially identifiable images or data included in this article.

## Author contributions

HN: Conceptualization, Data curation, Formal Analysis, Investigation, Methodology, Project administration, Software, Supervision, Validation, Visualization, Writing – original draft, Writing – review & editing. BL: Data curation, Formal Analysis, Investigation, Methodology, Software, Visualization, Writing – original draft, Writing – review & editing. GS: Data curation, Formal Analysis, Software, Visualization, Writing – original draft, Writing – review & editing. SK: Investigation, Methodology, Writing – review & editing. SR: Methodology, Writing – review & editing, Resources. MP: Software. JT: Software. DM: Methodology. LB: Formal Analysis, Visualization. AH: Conceptualization, Funding acquisition, Resources, Supervision – review & editing, Methodology.

## References

[1] McArdle CS Hole DJ. Outcome following surgery for colorectal cancer: analysis by hospital after adjustment for case-mix and deprivation. Br J Cancer (2002) 86:331–5. doi: 10.1038/sj.bjc.6600120 PMC2375219 11875693

[2] Haggar FA Boushey RP. Colorectal cancer epidemiology: incidence, mortality, survival, and risk factors. Clin Colon Rectal Surg (2009) 22:191–7. doi: 10.1055/s-0029-1242458 PMC2796096 21037809

[3] Dienstmann R Vermeulen L Guinney J Kopetz S Tejpar S Tabernero J. Consensus molecular subtypes and the evolution of precision medicine in colorectal cancer. Nat Rev Cancer (2017) 17:79–92. doi: 10.1038/nrc.2016.126 28050011

[4] Keum NN Giovannucci E. Global burden of colorectal cancer: emerging trends, risk factors and prevention strategies. Nat Rev Gastroenterol Hepatol (2019) 16:713–32. doi: 10.1038/s41575-019-0189-8 31455888

[5] Rutter MD Saunders BP Wilkinson KH Rumbles S Schofield G Kamm MA. Thirty-year analysis of a colonoscopic surveillance program for neoplasia in ulcerative colitis. Gastroenterology (2006) 130:1030–8. doi: 10.1053/j.gastro.2005.12.035 16618396

[6] Eaden JA Abrams KR Mayberry JF. The risk of colorectal cancer in ulcerative colitis: a meta-analysis. Gut (2001) 48(4):526–35. doi: 10.1136/gut.48.4.526 PMC1728259 11247898

[7] Ullman TA Itzkowitz SH. Intestinal inflammation and cancer. Gastroenterology (2011) 140:1807–1816.e1. doi: 10.1053/j.gastro.2011.01.057 21530747

[8] Picard E Verschoor CP Ma GW Pawelec G. Relationships between immune landscapes, genetic subtypes and responses to immunotherapy in colorectal cancer. Front Immunol (2020) 11:369. doi: 10.3389/fimmu.2020.00369 32210966 PMC7068608

[9] Pagès F Berger A Camus M Sanchez-Cabo F Costes A Molidor R. Effector memory T cells, early metastasis, and survival in colorectal cancer. N Engl J Med (2005) 353:2654–66. doi: 10.1056/NEJMoa051424 16371631

[10] Galon J Costes A Sanchez-Cabo F Kirilovsky A Mlecnik B Lagorce-Pagès C. Type, density, and location of immune cells within human colorectal tumors predict clinical outcome. Science (2006) 313:1960–4. doi: 10.1126/science.1129139 17008531

[11] Curtis NJ Primrose JN Thomas GJ Mirnezami AH Ottensmeier CH. The adaptive immune response to colorectal cancer: From the laboratory to clinical practice. Eur J Surg Oncol (2012) 38:889–96. doi: 10.1016/J.EJSO.2012.05.011 22721580

[12] Correale P Rotundo MS Botta C Del Vecchio MT Ginanneschi C Licchetta A. Tumor infiltration by T lymphocytes expressing chemokine receptor 7 (CCR7) is predictive of favorable outcome in patients with advanced colorectal carcinoma. Diagnosis (2012) 18(3):850–7. doi: 10.1158/1078-0432.CCR-10-3186 22142823

[13] Poeta VM Massara M Capucetti A Bonecchi R. Chemokines and chemokine receptors: New targets for cancer immunotherapy. Front Immunol (2019) 10:379. doi: 10.3389/fimmu.2019.00379 30894861 PMC6414456

[14] Deng H Unutmaz D Kewalramani VN Littman DR. Expression cloning of new receptors used by simian and human immunodeficiency viruses (1997). Available at: https://www.nature.com/articles/40894.pdf (Accessed April 29, 2019). 10.1038/40894 9230441

[15] Farzan M Choe H Martin K Marcon L Hofmann W Karlsson G. Two orphan seven-transmembrane segment receptors which are expressed in CD4-positive cells support simian immunodeficiency virus infection. J Exp Med (1997) 186:405–11. doi: 10.1084/jem.186.3.405 PMC2198994 9236192

[16] Unutmaz D KewalRamani VN Littman DR. G protein-coupled receptors in HIV and SIV entry: New perspectives on lentivirus-host interactions and on the utility of animal models. Semin Immunol (1998) 10:225–36. doi: 10.1006/smim.1998.0134 9653049

[17] Kim SV Xiang WV Kwak C Yang Y Lin XW Ota M. GPR15-mediated homing controls immune homeostasis in the large intestine mucosa. Sci (80- ) (2013) 340:1456–9. doi: 10.1126/science.1237013 PMC3762262 23661644

[18] Ocón B Pan J Dinh TT Chen W Ballet R Bscheider M. A mucosal and cutaneous chemokine ligand for the lymphocyte chemoattractant receptor GPR15. Front Immunol (2017) 8:1111. doi: 10.3389/fimmu.2017.01111 28936214 PMC5594226

[19] Suply T Hannedouche S Carte N Li J Grosshans B Schaefer M. A natural ligand for the orphan receptor GPR15 modulates lymphocyte recruitment to epithelia. Sci Signal (2017) 10(496):eaal0180. doi: 10.1126/scisignal.aal0180 28900043

[20] Fiocchi C Youngman KR. Isolation of human intestinal mucosal mononuclear cells. In: Current protocols in immunology. Hoboken, NJ, USA: John Wiley & Sons, Inc (2001). doi: 10.1002/0471142735.im0730s19 18432842

[21] Tang Z Li C Kang B Gao G Li C Zhang Z. GEPIA: A web server for cancer and normal gene expression profiling and interactive analyses. Nucleic Acids Res (2017) 45:W98–W102. doi: 10.1093/nar/gkx247 28407145 PMC5570223

[22] Li T Fu J Zeng Z Cohen D Li J Chen Q. TIMER2.0 for analysis of tumor-infiltrating immune cells. Nucleic Acids Res (2020) 48:W509–14. doi: 10.1093/nar/gkaa407 PMC7319575 32442275

[23] Lee HO Hong Y Etlioglu HE Cho YB Pomella V Van den Bosch B. Lineage-dependent gene expression programs influence the immune landscape of colorectal cancer. Nat Genet 2020 526 (2020) 52:594–603. doi: 10.1038/s41588-020-0636-z 32451460

[24] Neufert C Becker C Neurath MF. An inducible mouse model of colon carcinogenesis for the analysis of sporadic and inflammation-driven tumor progression. Nat Protoc (2007) 2:1998–2004. doi: 10.1038/nprot.2007.279 17703211

[25] Thaker AI Shaker A Rao MS Ciorba MA. Modeling colitis-associated cancer with azoxymethane (AOM) and dextran sulfate sodium (DSS). J Vis Exp (2012) 11(67):4100. doi: 10.3791/4100 PMC3490277 22990604

[26] Dieleman LA Palmen MJ Akol H Bloemena E Peña AS Meuwissen SG. Chronic experimental colitis induced by dextran sulphate sodium (DSS) is characterized by Th1 and Th2 cytokines. Clin Exp Immunol (1998) 114:385–91. doi: 10.1046/J.1365-2249.1998.00728.X PMC1905133 9844047

[27] Yim JJ Harmsen S Flisikowski K Flisikowska T Namkoong H Garland M. A protease-activated, near-infrared fluorescent probe for early endoscopic detection of premalignant gastrointestinal lesions. Proc Natl Acad Sci U.S.A. (2021) 118(1):e2008072118. doi: 10.1073/pnas.2008072118 33443161 PMC7817203

[28] Lefrançois L Lycke N. Isolation of mouse small intestinal intraepithelial lymphocytes, peyer’s patch, and lamina propria cells. In: Current protocols in immunology. Hoboken, NJ, USA: John Wiley & Sons, Inc (2001). doi: 10.1002/0471142735.im0319s17 18432783

[29] Loddenkemper C Schernus M Noutsias M Stein H Thiel E Nagorsen D. *In situ* analysis of FOXP3+ regulatory T cells in human colorectal cancer. J Transl Med (2006) 4:52. doi: 10.1186/1479-5876-4-52 17166272 PMC1764431

[30] Okayasu I Hatakeyama S Yamada M Ohkusa T Inagaki Y Nakaya R. A novel method in the induction of reliable experimental acute and chronic ulcerative colitis in mice. Gastroenterology (1990) 98:694–702. doi: 10.1016/0016-5085(90)90290-H 1688816

[31] Tanaka T Kohno H Suzuki R Yamada Y Sugie S Mori H. A novel inflammation-related mouse colon carcinogenesis model induced by azoxymethane and dextran sodium sulfate. Cancer Sci (2003) 94:965–73. doi: 10.1111/j.1349-7006.2003.tb01386.x PMC11160237 14611673

[32] Neufert C Heichler C Brabletz T Scheibe K Boonsanay V Greten FR. Inducible mouse models of colon cancer for the analysis of sporadic and inflammation-driven tumor progression and lymph node metastasis. Nat Protoc (2021) 16:61–85. doi: 10.1038/s41596-020-00412-1 33318692

[33] De Robertis M Massi E Poeta M Carotti S Morini S Cecchetelli L. The AOM/DSS murine model for the study of colon carcinogenesis: From pathways to diagnosis and therapy studies. J Carcinog (2011) 10:9. doi: 10.4103/1477-3163.78279 21483655 PMC3072657

[34] Parang B Barrett CW Williams CS. AOM/DSS model of colitis-associated cancer. Methods Mol Biol (2016) 1422:297–307. doi: 10.1007/978-1-4939-3603-8_26 27246042 PMC5035391

[35] Adamczyk A Pastille E Kehrmann J Vu VP Geffers R Wasmer MH. GPR15 facilitates recruitment of regulatory T cells to promote colorectal cancer. Cancer Res (2021) 81:2970–82. doi: 10.1158/0008-5472.CAN-20-2133/673664/AM/GPR15-FACILITATES-RECRUITMENT-OF-REGULATORY-T 33727229

[36] Barnes TA Amir E. HYPE or HOPE: The prognostic value of infiltrating immune cells in cancer. Br J Cancer (2017) 117:451–60. doi: 10.1038/bjc.2017.220 PMC5558691 28704840

[37] Corbett TH Griswold DP Roberts BJ Peckham JC Schabel FM. Tumor induction relationships in development of transplantable cancers of the colon in mice for chemotherapy assays, with a note on carcinogen structure. Cancer Res (1975) 35(9):2434–9. 1149045

[38] Efremova M Rieder D Klepsch V Charoentong P Finotello F Hackl H. Targeting immune checkpoints potentiates immunoediting and changes the dynamics of tumor evolution. Nat Commun (2018) 9(1):32. doi: 10.1038/s41467-017-02424-0 29296022 PMC5750210

[39] Taylor MA Hughes AM Walton J Coenen-Stass AML Magiera L Mooney L. Longitudinal immune characterization of syngeneic tumor models to enable model selection for immune oncology drug discovery. J Immunother Cancer (2019) 7(1):328. doi: 10.1186/s40425-019-0794-7 31779705 PMC6883640

[40] Lau J Cheung J Navarro A Lianoglou S Haley B Totpal K. Tumour and host cell PD-L1 is required to mediate suppression of anti-tumour immunity in mice. Nat Commun (2017) 8:14572. doi: 10.1038/ncomms14572 28220772 PMC5321797

[41] Nguyen LP Pan J Dinh TT Hadeiba H O’ E Iii H. Role and species-specific expression of colon T cell homing receptor GPR15 in colitis Europe PMC Funders Group. Nat Immunol (2015) 16:207–13. doi: 10.1038/ni.3079 PMC4338558 25531831

[42] Swaminathan G Nguyen LP Namkoong H Pan J Haileselassie Y Patel A. The aryl hydrocarbon receptor regulates expression of mucosal trafficking receptor GPR15. Mucosal Immunol (2021) 14:852–61. doi: 10.1038/S41385-021-00390-X PMC7934811 33674764

[43] Fujimoto H Saito Y Ohuchida K Kawakami E Fujiki S Watanabe T. Deregulated mucosal immune surveillance through gut-associated regulatory T cells and PD-1 + T cells in human colorectal cancer. J Immunol (2018) 200:3291–303. doi: 10.4049/jimmunol.1701222 29581358

[44] Guo F Wang Y Liu J Mok SC Xue F Zhang W. CXCL12/CXCR4: A symbiotic bridge linking cancer cells and their stromal neighbors in oncogenic communication networks. Oncogene (2016) 35:816–26. doi: 10.1038/onc.2015.139 25961926

[45] Bindea G Mlecnik B Tosolini M Kirilovsky A Waldner M Obenauf AC. Spatiotemporal dynamics of intratumoral immune cells reveal the immune landscape in human cancer. Immunity (2013) 39:782–95. doi: 10.1016/j.immuni.2013.10.003 24138885

[46] Tosolini M Kirilovsky A Mlecnik B Fredriksen T Mauger S Bindea G. Clinical impact of different classes of infiltrating T cytotoxic and helper cells (Th1, Th2, Treg, Th17) in patients with colorectal cancer. Cancer Res (2011) 71:1263–71. doi: 10.1158/0008-5472.CAN-10-2907 21303976

[47] Amicarella F Muraro MG Hirt C Cremonesi E Padovan E Mele V. Dual role of tumour-infiltrating T helper 17 cells in human colorectal cancer. Gut (2017) 66:692–704. doi: 10.1136/gutjnl-2015-310016 26719303 PMC5529969

[48] Fischer A Zundler S Atreya R Rath T Voskens C Hirschmann S. Differential effects of α4β7 and GPR15 on homing of effector and regulatory T cells from patients with UC to the inflamed gut. vivo. Gut (2016) 65:1642–64. doi: 10.1136/gutjnl-2015-310022 PMC5036234 26209553

[49] Adamczyk A Gageik D Frede A Pastille E Hansen W Rueffer A. Differential expression of GPR15 on T cells during ulcerative colitis. JCI Insight (2017) 2(8):e90585. doi: 10.1172/JCI.INSIGHT.90585 28422750 PMC5396530

[50] Krijgsman D Hokland M Kuppen PJK. The role of natural killer T cells in cancer-A phenotypical and functional approach. Front Immunol (2018) 9:367. doi: 10.3389/fimmu.2018.00367 29535734 PMC5835336

[51] Pan W Cheng Y Zhang H Liu B Mo X Li T. CSBF/C10orf99, a novel potential cytokine, inhibits colon cancer cell growth through inducing G1 arrest. Sci Rep (2014) 4:6812. doi: 10.1038/srep06812 25351403 PMC4212244

[52] Hayn M Blötz A Rodríguez A Vidal S Preising N Ständker L. Natural cystatin C fragments inhibit GPR15-mediated HIV and SIV infection without interfering with GPR15L signaling. Proc Natl Acad Sci U.S.A. (2021) 118(3):e2023776118. doi: 10.1073/pnas.2023776118 33431697 PMC7826402

[53] Wang Y Wang X Xiong Y Li CD Xu Q Shen L. An integrated pan-cancer analysis and structure-based virtual screening of GPR15. Int J Mol Sci (2019) 20(24):6226. doi: 10.3390/ijms20246226 31835584 PMC6940937

[54] Jiang D Xie X Lu Z Liu L Qu Y Wu S. Establishment of a colorectal cancer-related microRNA-mRNA regulatory network by microarray and bioinformatics. Front Genet (2020) 11:560186. doi: 10.3389/fgene.2020.560186 33193642 PMC7644864

[55] Guo Y Zhu Q Chen S Li Y Fu D Qiao D. Post-transcriptional suppression of G protein-coupled receptor 15 (GPR15) by microRNA-1225 inhibits proliferation, migration, and invasion of human colorectal cancer cells. 3 Biotech (2021) 11:139. doi: 10.1007/S13205-021-02682-2 PMC7902748 33708462

[56] Masuda K Kornberg A Miller J Lin S Suek N Botella T. Multiplexed single-cell analysis reveals prognostic and nonprognostic T cell types in human colorectal cancer. JCI Insight (2022) 7(7):e154646. doi: 10.1172/JCI.INSIGHT.154646 35192548 PMC9057629

[57] Sackstein R Schatton T Barthel SR. T-lymphocyte homing: An underappreciated yet critical hurdle for successful cancer immunotherapy. Lab Investig (2017) 97:669–97. doi: 10.1038/labinvest.2017.25 PMC5446300 28346400

